# WSC-1 and HAM-7 Are MAK-1 MAP Kinase Pathway Sensors Required for Cell Wall Integrity and Hyphal Fusion in *Neurospora crassa*

**DOI:** 10.1371/journal.pone.0042374

**Published:** 2012-08-03

**Authors:** Abhiram Maddi, Anne Dettman, Ci Fu, Stephan Seiler, Stephen J. Free

**Affiliations:** 1 Department of Biological Sciences, State University of New York, University at Buffalo, Buffalo, New York, United States of America; 2 Department of Periodontics and Endodontics, School of Dental Medicine, State University of New York, University at Buffalo, Buffalo, New York, United States of America; 3 Institute for Microbiology and Genetics, Department of Molecular Microbiology and Genetics, Georg-August-University, Göttingen, Germany; Rutgers University, United States of America

## Abstract

A large number of cell wall proteins are encoded in the *Neurospora crassa* genome. Strains carrying gene deletions of 65 predicted cell wall proteins were characterized. Deletion mutations in two of these genes (*wsc-1* and *ham-7*) have easily identified morphological and inhibitor-based defects. Their phenotypic characterization indicates that HAM-7 and WSC-1 function during cell-to-cell hyphal fusion and in cell wall integrity maintenance, respectively. *wsc-1* encodes a transmembrane protein with extensive homology to the yeast Wsc family of sensor proteins. In *N. crassa*, WSC-1 (and its homolog WSC-2) activates the cell wall integrity MAK-1 MAP kinase pathway. The GPI-anchored cell wall protein HAM-7 is required for cell-to-cell fusion and the sexual stages of the *N. crassa* life cycle. Like WSC-1, HAM-7 is required for activating MAK-1. A Δ*wsc-1;*Δ*ham-7* double mutant fully phenocopies mutants lacking components of the MAK-1 MAP kinase cascade. The data identify WSC-1 and HAM-7 as the major cell wall sensors that regulate two distinct MAK-1-dependent cellular activities, cell wall integrity and hyphal anastomosis, respectively.

## Introduction

The fungal cell wall is a dynamic structure that functions to communicate with and to protect the cell from the environment. Its basic structure consists of an interconnected network of glucan and chitin polymers [1], [2], [3], [4], [5], [6]. These polymers are synthesized by plasma membrane-associated α- and β-glucan synthases and chitin synthases, and extruded into the cell wall space during synthesis. These linear polymers are then cross-linked by a collection of glucanases, chitinases and glycosyltransferases to generate the mature cell wall matrix. Most cell wall proteins are synthesized on ER-associated polysomes and pass through the secretory pathway, where they are extensively modified by the addition of N-linked and O-linked oligosaccharides. About half of the identified *Neurospora crassa* cell wall proteins have glycosylphosphatidylinositol (GPI) anchors at their carboxyl terminus that are used to attach the proteins to the extracellular leaflet of the plasma membrane [7], [8], [9]. Integral cell wall proteins are covalently cross-linked into the glucan/chitin matrix.

The cell wall is a dynamic structure and its’ composition is responsive to changes in environmental conditions. Some cell wall proteins function as sensors for signal transduction pathways, and allow the cell to assess and respond to changing environments [2], [3], [10], [11]. The best-characterized pathway that monitors cell wall stress is the cell wall integrity (CWI) pathway. This pathway includes cell wall sensors, the plasma membrane-associated small GTPase Rho1, protein kinase C (Pkc1), and a mitogen-activated protein (MAP) kinase cascade. Activation of the CWI pathway induces the expression of cell wall proteins to maintain cell wall integrity [2], [10], [12]. In *S. cerevisiae*, a family of five membrane-spanning sensors (Wsc1p, Wsc2p, Wsc3p, Mid1p and Mtl1p) activate the CWI pathway [12], [13], [14]. It was shown for Wsc1p that the cytoplasmic C terminal domain activates Rho1p through interactions with its GTP exchange factor Rom2p. In *N. crassa*, the MIK-1/MEK-1/MAK-1 MAP kinase cascade functions as the cell wall integrity signaling system, and is required for normal hyphal growth, colony morphology, and sexual development [15], [16]. Moreover, the MAK-1 pathway has also been implicated in cell-cell signaling and hyphal anastomosis [15], [17], [18]. However, its precise function in self-signaling and its functional relationship with MAK-2, a second MAP kinase cascade that plays a critical role in hyphal anastomosis, is unknown. In contrast to MAK-1, MAK-2 module is recruited in an oscillatory fashion to the tips of fusion hyphae, and thus is thought to constitute the critical MAP kinase pathway that directs the cell fusion process [15], [19], [20], [21].

In an effort to identify functions that can be ascribed to individual cell wall proteins, we characterized 65 gene deletion mutants lacking predicted cell wall proteins. Mutants affecting most of these proteins are resistant to cell wall stress reagents and have normal morphology, indicating that they have fully functional cell walls. This suggests that there might be a large level of functional redundancy among the cell wall proteins. We identified two cell wall protein genes, *wsc-1* and *ham-7*, that are required for normal vegetative cell morphology and resistance to stress reagents. *N. crassa* WSC-1 (and its homolog WSC-2) is required for the activation of the CWI MAK-1 MAP kinase pathway. The second gene, *ham-7*, was previously identified as required for cell-to-cell fusion and sexual development [22]. We show that HAM-7 is also needed for the activation of the *N. crassa* MAK-1 MAP kinase pathway. Our data suggest that WSC-1 and HAM-7 are cell wall sensors which function to control two distinct cellular activities of the MAK-1 signal transduction pathway, CWI and cell-to-cell fusion respectively.

## Materials and Methods

### Identification of Genes Encoding Cell Wall Proteins and Mutants Lacking these Cell Wall Proteins

*N. crassa* cell wall proteins were identified by three methods. First, cell wall proteins were identified from a proteomic analysis of purified vegetative and conidial cell walls [8], [9]. Second, *N. crassa* GPI-anchored proteins were identified by bioinformatics approaches [23], [24] and were assumed to be cell wall proteins. Third, the published *N crassa* genome sequence [25] was used to identify potential homologs of known cell wall proteins from *S. cerevisiae*, *Schizosaccharmyces pombe*, *C. albicans*, and *Aspergillus* species by homology searches. The list of potential *N. crassa* cell wall proteins was then used to search the single gene deletion library prepared by the Neurospora genome project [26], [27], [28]. Using this approach we identified 65 cell wall protein genes for which deletion mutants were available (Table S1).

### Growth of the Mutants and Screening Procedures to Identify Mutant Phenotypes

Cells were routinely grown and maintained on 3 ml slants of Vogel’s agar medium supplemented with 2% sucrose as described by Davis and De Serres [29]. Screening for morphological characteristics associated with the asexual phase of the life cycle was carried out by examination of the colony morphology during growth on Vogel’s sucrose agar medium. Examination of the mutants growing on synthetic crossing medium was used to determine whether they were affected in the formation of protoperithecia (immature female mating structures). Protoperithecia were fertilized with wild type conidia of the opposite mating type, and the ability of the protoperithicia to complete female development and produce ascospores was assessed.

The growth rate is affected by a large range of cell wall unrelated mutations [1], [7], [30] and thus, we did not use a reduced growth rate as criterium for a cell wall defect. However, growth rate measurements at 30°C were made on mutants that had been identified as having altered morphologies and mutants that were susceptible to cell wall stress reagents. The diameter of the vegetative hyphae at the edge of a colony was determined by taking photographs of the hyphae, measuring the diameter of 20 hyphae, and averaging the measured values.

### Stress Tests

The growth of the 65 deletion mutants in the presence of the various stress reagents was observed at 30°C over a period of 72 hours and compared to the growth of the wild type. The concentrations of the stress reagents used were determined by subjecting the wild type strain to a range of concentrations and identifying a concentration which was slightly below the concentration at which the wild type cells were unable to grow [31]. Stress reagents included 10% NaCl (salt stress), 2 M glycerol (hyperosmolarity), 0.05 mM hydrogen peroxide (oxidative stress), 0.01% SDS (detergent), 10 mg/ml Calcoflour white (chitin synthesis inhibitor; Sigma-Aldrich Chemical Company, St. Louis, MO), and 10 µg/ml caspofungin acetate (glucan synthesis inhibitor; obtained as a kind gift from Merck Research Laboratories; Rahway, NJ).

### Genetic Co-segregation Experiments

The deletion mutants found in the *N. crassa* single gene deletion library were generated by replacing the coding region of the genes with a hygromycin cassette [26]. We have found that many of the mutants in the library have mutations in addition to the deletion mutation [22]. Thus, it was necessary to verify that the mutant phenotype co-segregated with the deletion mutation. Co-segregation experiments were carried out by mating each of the mutants with the wild type strain of the opposite mating type as described by Davis and De Serres [29]. At least 24 individual progeny from each of the crosses were isolated and tested for the presence or absence of the mutant phenotype using the same criteria used in the mutant screening procedures mentioned above. Each of the progeny was also tested for the presence of the deletion mutation by assessing the ability of the progeny to grow in the presence of 200 µg/ml hygromycin. Co-segregation of the hygromycin resistance with the mutant phenotype and hygromycin sensitivity with the wild type phenotype was taken as evidence that the deletion mutation was responsible for the mutant phenotype.

### Complementation Analysis

The ability of a wild type copy of the gene to complement the deletion phenotype was used to test if the mutant characteristics were due to the gene deletion. The pBM60/pBM61 vector system designed by Margolin et al. [32] was used for these complementation experiments. These plasmids contain wild type *his-3* sequences and allow for the site-directed insertion of the cloned DNA in the intergenic region 3′ of the *his-3* locus. The deletion mutants were first mated with a *his-3* mutant (allele Y234M723*)* of the opposite mating types and mutant progeny carrying both the deletion mutation (assessed by the presence of the mutant phenotype and by hygromycin resistance) and the *his-3* mutation were isolated. For each of the mutant genes, a plasmid vector derived from the pBM60 or pBM61 plasmid containing a wild type copy of the genomic DNA from approximately 1,500 base pairs of sequence upstream of the coding region to 500 base pairs downstream of the coding region was prepared. The genes were amplified by PCR from wild type genomic DNA using primers that had restriction sites added at their 5′ and 3′ ends to facilitate cloning of the PCR products. The primers used for these cloning experiments are provided in Table S2. The plasmids containing the cloned genes were sequenced to verify that the cloned gene had a wild type sequence. The plasmids were then used in transformation experiments with conidia from a *his-3;* Δ*ham-7, his-3;* Δ*acw-4,* and *his-3;* Δ*wsc-1* double mutants. During the insertion event, a wild type copy of the *his-3* gene is generated, which allows the transformants to be selected for on a non-supplemented medium. To render the transformants homokaryotic, transformant conidia were streaked onto sorbose plates and single conidial isolates were isolated. This process of single conidial isolation was repeated five times for the transformants. The presence of the original deletion mutations and the insertion of the wild type copy of the genes were verified by PCR using primers that allowed for the amplication of the gene deletion and insertion genomic sequences.

**Figure 1:**
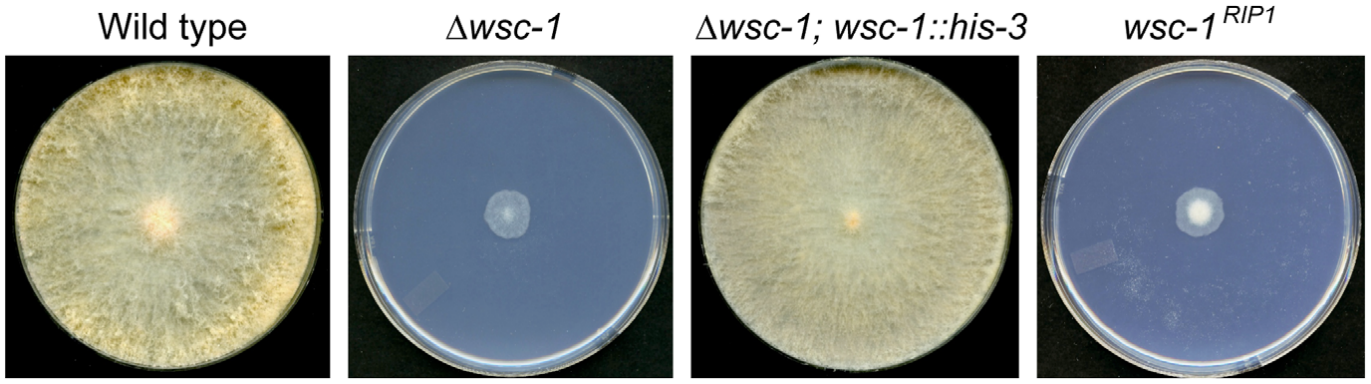
Complementation and RIP analysis of *wsc-1*. Colony morphologies are shown for wild type, Δ*wsc-1*, a transformant of Δ*wsc-1* containing a wild type copy of the *wsc-1* gene inserted at the *his-3* locus, and the *wsc-1^RIP1^* mutant grown for 24 hours on a Vogel’s sucrose agar medium.

### RIP Mutational Analysis

During the sexual cycle of *N. crassa*, isolates in which a gene is present in duplicate copies in the haploid genome participate in the RIP (Repeat Induced Point mutation) phenomenon. During RIP, multiple C to T mutations are generated in both copies of the duplicated DNA [33]. We used RIP to demonstrate that a mutated allele of *wsc-1* phenocopied the deletion strain. We transformed a *his-3* isolate (allele Y234M723) with a wild type copy of the *wsc-1* gene in the pBM61 plasmid as described above. To activate the RIP process, the transformants were mated with a *his-3* isolate of the opposite mating type. In the process of a genetic cross, RIP generates mutations in the endogenous and transforming copies of the cell wall genes. Individual progeny from these *his-3::wsc-1* x *his-3* crosses were then characterized. *his-3^−^* progeny with a phenotype corresponding to Δ*wsc-1* were identified, and the endogenous copy of the *wsc-1* was amplified by PCR and sequenced.

### Activation of the MAK-1 and MAK-2 Signal Transduction Pathways

Liquid *N. crassa* cultures were grown at room temperature, harvested gently by filtration using a Büchner funnel and ground in liquid nitrogen. Oxidative stress was induced by addition of 8 mM H_2_O_2_ for 10 min. Protein extraction for the analysis of the MAK1 and MAK2 phosphorylation status was performed as described [34], [35], [36] with minor modifications. Briefly, the frozen mycelial powder was incubated in 95% ethanol at −20°C for ≥12 h, the supernatant removed after centrifugation and the pellet vacuum-dried in a SpeedVac concentrator (Thermo Fisher Scientific, USA). Extraction buffer (100 mM Tris pH7.0, 1% (w/v) SDS; supplemented with 5 mM NaF, 1 mM PMSF, 1 mM Na3VO4, 25 mM β-glycerophosphate, 2 mM benzamidine, 2 ng/µl pepstatin A, 10 ng/µl aprotinin, 10 ng/µl leupeptin) was added, the samples mixed and incubated at 80°C for 5 min and the supernatant collected after centrifugation. After a second round of extraction, the supernatants were pooled, subjected to another centrifugation step, and the protein concentration determined using a Nanodrop spectrophotometer (ND-1000, Peqlab, Germany). Sample volumes corresponding to 75 µg total protein per lane were subjected to SDS polyacrylamide gel electrophoresis and subsequent Western blotting using polyclonal rabbit α-Phospho-p44/42 MAPK (Cell Signaling Technology, Inc., USA) and goat α-rabbit IgG-HRP (Santa Cruz, USA) as primary and secondary antibodies, respectively. For quantification of MAK1 and MAK2 phosphorylation levels, exposed films were scanned at a resolution of 600 dpi and densitometry was performed on the resulting tiff-files employing the AIDA Image Analyzer (version 4.22; raytest Isotopenmessgeräte, Germany) in transmission mode. Intensity values [arbitrary units] measured within a region of interest of fixed size containing the MAK1 or MAK2 protein bands were corrected by subtraction of local background, normalized to the protein amount loaded and used for further evaluation.

**Table 1:** Mutant Phenotypes.

Gene Name (NCU#)	Protein function and size	Sensitivity to stress reagents	Phenotypic characterization
Wild type		none	Wild type
**Δ** ***mak-1*** ** (NCU09842)**	MAPK	Caspofungin; Calcofluor White	- compact growth
			- rosetta-like colony
			- very poor aerial hyphae formation
			- almost aconidial
			- cell fusion defective
			- no protoperithecia
**Δ** ***mek-1*** ** (NCU06419)**	MAPKK	Caspofungin; Calcofluor White	- identical to Δ*mak-1*
**Δ** ***ham-7*** ** (NCU00881)**	229 aa protein; GPI anchoredcell wall sensor	none	- altered growth and branching pattern
			- reduced formation of aerial hyphae
			- de-repressed conidiation
			- no protoperithecia
			- cell fusion defective
**Δ** ***wsc-1*** ** (NCU06910)**	300 aa proteintransmembrane sensor	Caspofungin; Calcofluor White	- compact growth
			- very poor aerial hyphae formation
			- almost aconidial
***wsc-1^RIP1^***	300 aa proteintransmembrane sensor	Caspofungin; Calcofluor White	- identical to Δ*wsc-1*
**Δ** ***wsc-1;*** **Δ** ***ham-7***		Caspofungin; Calcofluor White	- compact growth
			- rosetta-like colony
			- very poor aerial hyphae formation
			- almost aconidial
			- cell fusion defective
			- no protoperithecia

## Results

### Identification of Cell Wall Protein Mutants with Easily Identified Phenotypes

As described in Materials and Methods, predicted cell wall protein genes were identified by a combination of (i) cell wall proteomic analysis, (ii) bioinformatics programs that identified GPI-anchored proteins, and (iii) identifying *N. crassa* homologs for cell wall proteins found in other fungi. Deletion strains for 65 of these predicted cell wall protein genes were identified in the *N. crassa* gene deletion library (Table S1). In order to determine potential functions provided by these proteins, the deletion mutants were screened for morphological and cell wall defects using a number of cell wall inhibitor assays and stress tests. We identified 10 mutants that displayed either clear morphological defects or various stress-induced phenotypes (Table S3). Many of the 65 deletion mutants were gene deletions for cell wall enzymes that are thought to function in the creation of the cross-linked glucan/chitin matrix. None of these strains had easily observed defects. Most of the respective proteins are found in multi-gene families, suggesting that the encoded enzymes have overlapping enzymatic activities.

The gene deletion library was generated as part of the Neurospora Genome Project [26], [27]. During the construction of the deletion mutants, the genes being deleted were replaced with a hygromycin-resistance cassette. However, secondary mutations in other genes often arise during the procedure [22], and thus, we carried out co-segregation experiments as an initial test to determine, if the mutant phenotype was due to the deletion mutation. Each of the 10 mutants was mated with the wild type strain of the opposite mating type and the segregation of the hygromycin-resistance cassette and the mutant phenotype into the progeny was followed. We found that for three deletion mutants (*acw-4/NCU09263*, *ham-7/NCU00881*, and *wsc-1/NCU06910*), the hygromycin-resistance and mutant phenotypes co-segregated (Table S3), suggesting that the deletion mutations might be responsible for the mutant phenotype. For 7 of the 10 mutants, the mutant phenotype and the hygromycin-resistance did not co-segregate, indicating that the deletion mutations were not responsible for the mutant phenotypes in these strains (Table S3).

**Figure 2:**
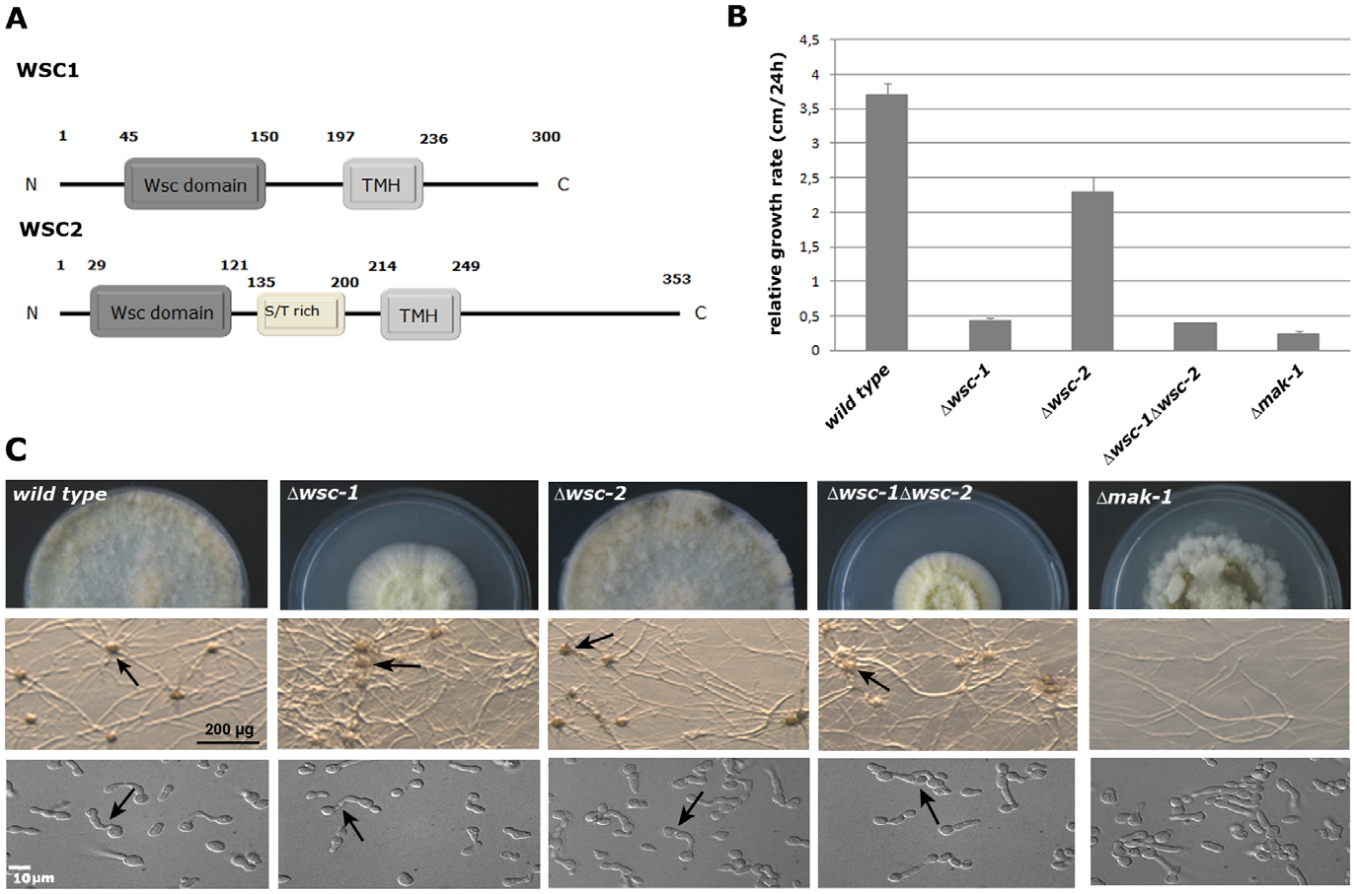
Characteristics of the Δ*wsc-1* and Δ*wsc-2* mutants. (**A**) The organization of the WSC-1 and WSC-2 proteins includes a N terminal extracellular region with a WSC domain (and a serine/threonine-rich region in WSC-2), a transmembrane helical sequence (TMH), and an intracellular carboxyl terminus. (**B**) Growth rates for wild type, Δ*wsc-1*, Δ*wsc-2*, Δ*wsc-1;* Δ*wsc-2*, and Δ*mak-1* are shown. (**C**) Images of the colony morphology (upper panel), the production of protoperithecia on Cornmeal agar (middle panel), and CAT fusion (lower panel) for wild type, Δ*wsc-1*, Δ*wsc-2*, Δ*wsc-1;* Δ*wsc-2*, and Δ*mak-1* are shown. The arrows in the middle and lower panels point to protoperithecia and CAT fusions.

### Complementation Experiments and RIP Mutational Analysis Demonstrate that *wsc-1* and *ham-7* Deletion Mutations are Responsible for the Mutant Phenotypes

Complementation experiments were used to verify that the deletion mutations were responsible for the mutant phenotypes. We had previously demonstrated that the Δ*ham-7* defects can be fully complemented by a wild type copy of the *ham-7* gene [22]. Transformation of Δ*wsc-1* with a wild type copy of *wsc-1* restored the wild type phenotype, demonstrating that the mutant characteristics were due to the loss of *wsc-1* (Figure 1). In similar complementation experiments, we isolated 16 independent transformants of Δ*acw-4* transformed with a wild type copy of *acw-4,* and found that the wild type copy of *acw-4* did not complement the mutant phenotype. We further characterized one of the transformants and demonstrated that it contained a wild type copy of *acw-4*. We concluded that the mutant phenotype was not due to the *acw-4* deletion.

In an alternative approach to verify that loss of *wsc-1* is responsible for the mutant phenotype, we generated additional mutant alleles of the *wsc-1* using RIP and compared them with the deletion strain. We found that *wsc-1^RIP^* mutants were indistinguishable from Δ*wsc-1* (Figure 1). We sequenced four of the *wsc-1^RIP^* alleles and verified that in all cases the gene had been inactivated by RIP mutations (GenBank accession #’s JQ520130 to JQ520133). We confirmed that the predicted sequence of *wsc-1* is correct, that the gene has two introns, and encodes a 300 amino acid protein. The first sequenced allele, *wsc-1^RIP1^*, had 84 mutations in the coding region, including a mutated start of translation codon and 4 stop codons. The second sequenced allele, *wsc-1^RIP2^*, had 4 mutations in the coding region, including a stop codon at amino acid #91 and a mutation that disrupts the intron/exon boundary of the second intron. The third allele, *wsc-1^RIP3^*, had 77 mutations in the coding region, including 5 stop codons, the first of which occurs at amino acid #35. The final sequenced allele, *wsc-1^RIP4^*, had 131 mutations in the coding region, including a mutated start codon and 8 stop codons. From the numbers and locations of these mutations, we conclude that these alleles are unable to make functional WSC-1, confirming that the defects observed for Δ*wsc-1* are due to the deletion of *wsc-1*.

### Δ*wsc-1* is Affected in Cell Wall Integrity and Asexual Development

To further characterize Δ*wsc-1*, the mutant was subjected to a variety of stress reagents and to a morphological characterization. Δ*wsc-1* was sensitive to presence of the glucan inhibitor caspofungin and the chitin synthesis inhibitor calcofluor white, indicating that the mutant has a cell wall defect (Table 1). Δ*wsc-1* also had alterations in its vegetative growth pattern, and displayed strong morphological defects during asexual development (Figures 1 and 2). Δ*wsc-1* grew very slowly, with a spreading colonial morphology, and a major reduction in the formation of aerial hyphae and conidia. These phenotypes are similar to mutants defective in components of the *mak-1* CWI MAP kinase cascade [15], [16], [37], suggesting that WSC-1 may function in regulating cell wall biogenesis through the MAK-1 pathway. However, MAK-1 kinase cascade mutants are also cell fusion defective and unable to produce female mating structures, called protoperithecia, while Δ*wsc-1* was cell fusion competent and able to go through the sexual phases of the *N. crassa* life cycle (Figure 2; Table 1). This suggested that the canonical CWI pathway may not be involved in intercellular communication and sexual development.

*wsc-1* encodes a 300 amino acid protein with a putative signal peptide at the N-terminus and an extracellular domain of ca. 200 amino acids, followed by a predicted transmembrane domain and a C-terminal intracellular region of ca. 70 amino acids (Figure 2A). Sequence analysis of WSC-1 indicates that it has protein sequence homology to the yeast Wsc1p, Wsc2p, and Wsc3p proteins, which function as sensors for the yeast CWI pathway [13]. The homology between *N. crassa wsc-1* and the yeast sensors was particularly apparent in the intracellular C-terminal region, which functions to activate the CWI signal transduction pathway through interaction with the Rho1p GTPase module. In addition to *wsc-1*, the *N. crassa* genome harbors a second gene with similar domain structure, which we designated *wsc-2* (NCU06981). In contrast to the Δ*wsc-1* deletion strain, Δ*wsc-2* had a phenotype close to wild type and displayed only a slightly reduced growth rate. We also noticed a slight reduction in the formation of conidia (Figure 2B, 2C). We generated a Δ*wsc-1;* Δ*wsc-2* double mutant, which phenocopied the Δ*wsc-1* deletion defects, indicating that WSC-1 is the major cell wall sensor in *N. crassa*. However, the Δ*wsc-1* and Δ*wsc-1;* Δ*wsc-2* double mutant defect did not fully resemble the rosetta-like vegetative growth phenotype of a Δ*mak-1* deletion strain and the fact Δ*wsc-1;* Δ*wsc-2* was still fusion competent and female fertile further indicated that additional MAK-1 signals are received through WSC-independent sensors.

**Figure 3:**
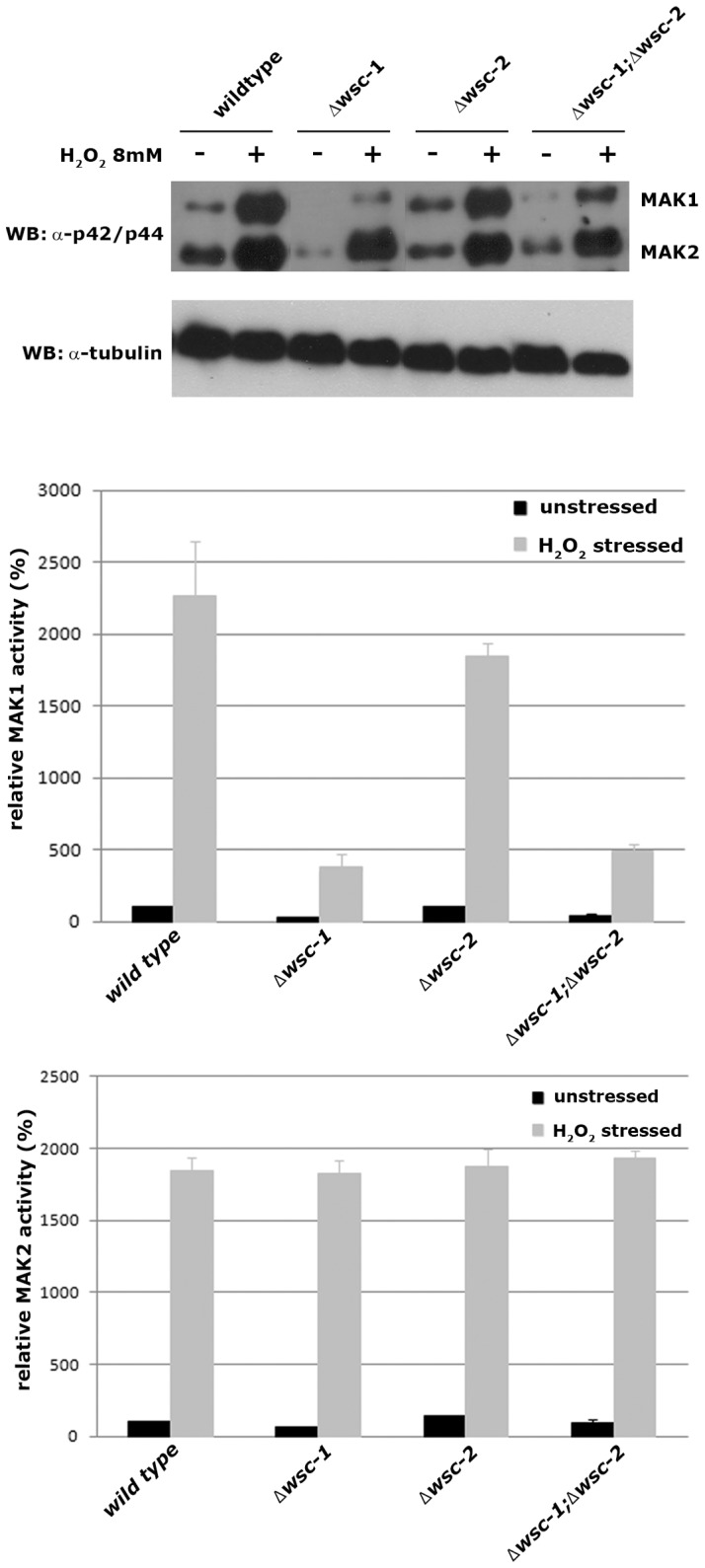
The Δ*wsc-1* mutant is deficient in MAK-1 activation. In the upper panel, extracts of non-stressed and oxidatively stressed wild type, Δ*wsc-1*, Δ*wsc-2*, and Δ*wsc-1;* Δ*wsc-2* cells were prepared and assayed for the presence of phosphorylated MAK-1 and MAK-2 by a Western blot assay using antibody that specifically recognizes the phosphorylated proteins. Extracts from non-stressed and from stressed cells are denoted by – and +. The sizes of the MAK-1 and MAK-2 proteins are shown at the side of the Western blot. A Western blot against tubulin was used as a control and to calibrate the amounts of protein in each of the samples. The amounts of activated MAK-1 (middle panel) and MAK-2 (lower panel) in each of the samples relative to the amount of activated MAK-1 or MAK-2 in the non-stressed wild type cell are shown (n = 5).

### WSC-1 and WSC2 Regulate MAK-1 Activity

The protein organization of WSC-1 and WSC-2, and the phenotypes of Δ*wsc-1* and the Δ*wsc-1;* Δ*wsc-2* double mutant strongly suggest that the two proteins are cell wall sensors for the CWI pathway. To determine whether WSC-1 and WSC-2 function upstream of MAK-1, the activity status of MAK-1 in the three *wsc* strains was assayed (Figure 3). The activity of MAK-2, the second ERK-type MAP kinase present in *N. crassa* that seems the primary signaling module regulating vegetative cell fusion [18], [19] was determined as control. In wild type, MAK-1 and MAK-2 display basal activities that can be stimulated ca. 20-fold under conditions of oxidative stress [15]. The basal and stress-induced MAK-2 activity levels were close to normal in Δ*wsc-1*, Δ*wsc-2* and the double mutant, and we detected only slightly reduced MAK-2 levels in Δ*wsc-1* (67%+/−15%; n = 5). We conclude that the MAK-2 pathway is fully functional in the *wsc* mutants, consistent with the observed cell fusion competence in the three strains (Table 1; Figure 2B, 2C). In contrast, basal MAK-1 activity of Δ*wsc-1* was reduced to 33%+/−12% (n = 5) and stress-induction of the MAP kinase was poor. As expected from its near wild type morphology, basal MAK-1 activity levels were normal in Δwsc-2 and stress-induced activity only slightly reduced. The MAK-1 activity levels in the Δ*wsc-1;* Δ*wsc-2* double mutant was comparable to the Δ*wsc-1* single mutant. We conclude that WSC-1 and WSC2 are required for activation of the *N. crassa* MAK-1 MAP kinase pathway, and that the WSC proteins function as extracellular sensors of the CWI pathway.

### Δham-7 is Affected in Cell-to-cell Fusion and Female Development

**Figure 4:**
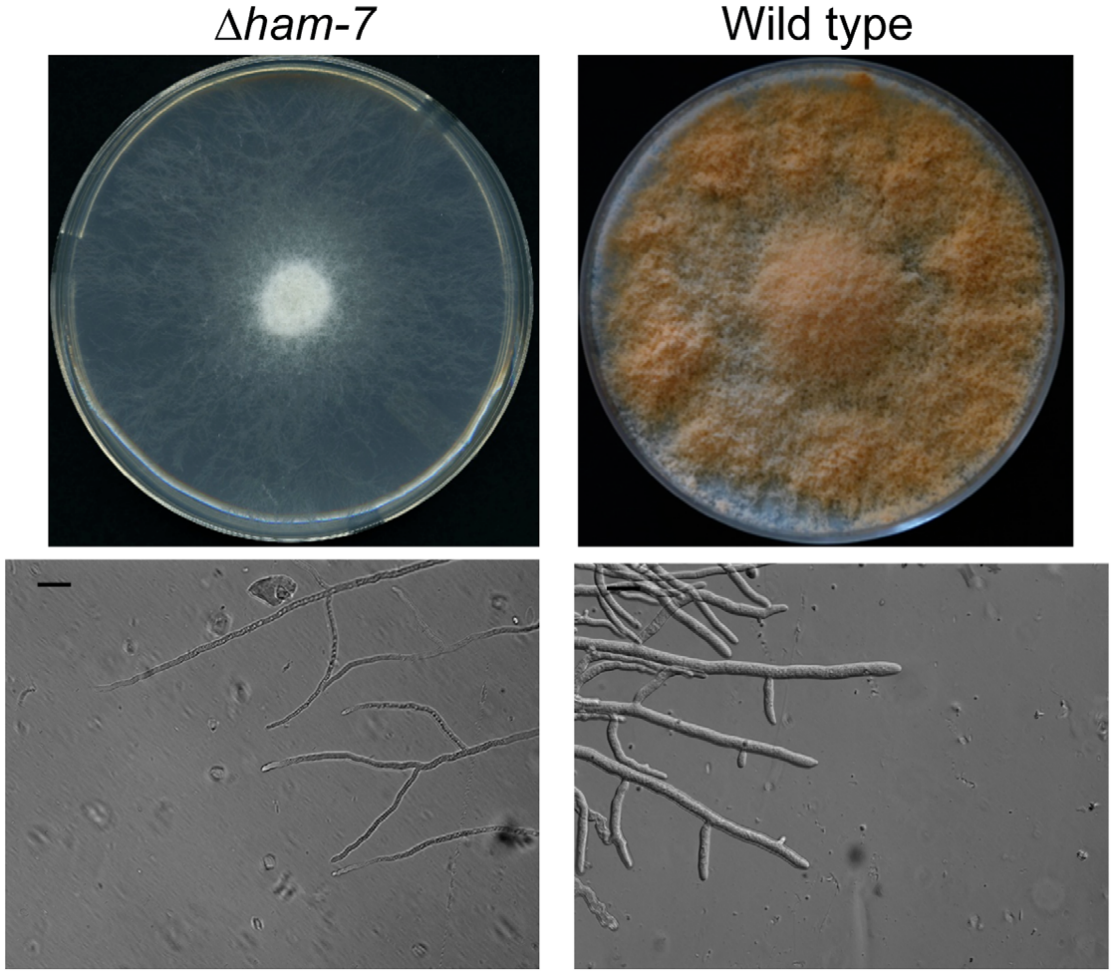
Δ*ham-7* morphologies. In the upper panels, the colonial morphology of Δ*ham-7* and wild type are shown for cells grown on a Vogel’s sucrose agar medium for 48 hours. The morphology of the hyphae at the edge of a 24 hour colony growing between two sheets of cellophane is shown for wild type and mutant cells in the lower panels. The black bar represents a distance of 20 µm.

The *ham-7* gene encodes a 229 amino acid protein with a typical signal peptide at the N-terminus and a GPI-anchor signal at the C–terminus. Thus, HAM-7 is an extracellular protein that is attached to the outer leaflet of the plasma membrane though its GPI-anchor. Δ*ham-7* was not sensitive to any of the cell wall stress reagents tested (Table 1). Δ*ham-7* displayed a de-repressed conidiation pattern and produced conidia on short aerial hyphae over the entire surface of the slant or Petri dish (Figure 4). The mutant clearly differed from wild type and Δ*wsc-1* in hyphal diameter and hyphal branching pattern. The diameter of Δ*ham-7* hyphae was 3.9+/−0.6 µm while the wild type hyphae had a diameter of 7.5+/−0.9 µm. Δ*ham-7* was also unable to generate protoperithecia, the *N. crassa* female mating structures. A “flattened” conidiation pattern and defective protoperithecium formation are typical characteristics of *N. crassa* cell fusion mutants [34], [38], [39], [40], [41]. Δ*ham-7* was previously identified in a mutant screen designed to identify hyphal anastomosis mutants and shown to be unable to participate in cell-to-cell fusion [22].

### HAM-7 Functions in Regulating MAK-1

**Figure 5:**
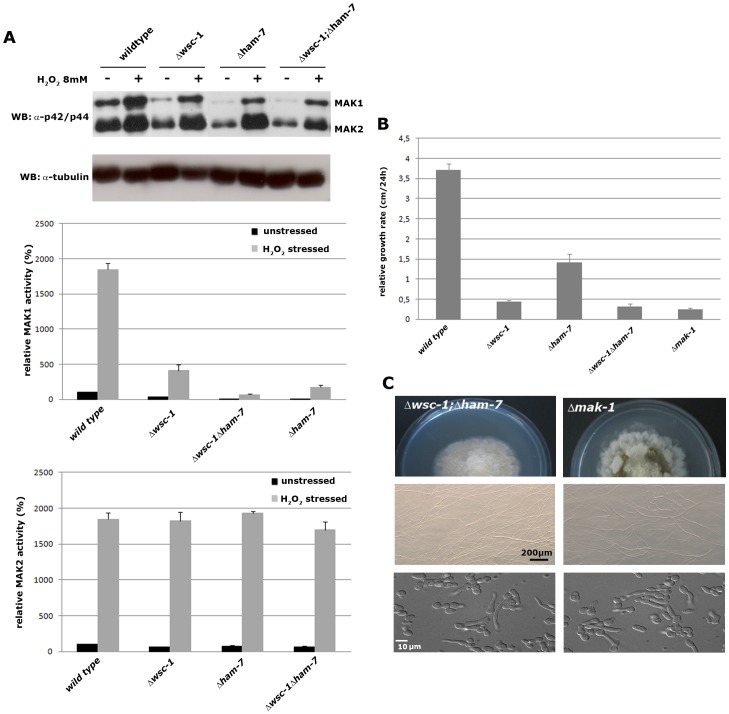
Δ*wsc-1*, Δ*ham-7*, and Δ*wsc-1;* **Δ** ***ham-7*** **are deficient in MAK-1 activation.** (**A**) In the upper panel, extracts of non-stressed and oxidatively stressed wild type, Δ*wsc-1*, Δ*ham-7*, and Δ*wsc-1;* Δ*ham-7* cells were prepared and assayed for the presence of phosphorylated MAK-1 and MAK-2 by a Western blot assay using antibody that specifically recognizes the phosphorylated proteins. Extracts from non-stressed and from stressed cells are denoted by – and +. The sizes of the MAK-1 and MAK-2 proteins are shown at the side of the Western blot. A Western blot against tubulin was used as a control and to calibrate the amounts of protein in each of the samples. The amounts of activated MAK-1 (middle panel) and MAK-2 (lower panel) in each of the samples relative to the amount of activated MAK-1 or MAK-2 in the non-stressed wild type cell are shown (n = 5). (**B**) The colony extension rates for wild type, Δ*wsc-1*, Δ*ham-7*, Δ*wsc-1;* Δ*ham-7*, and Δ*mak-1* are shown. (**C**) The colony morphology (upper panel), the lack of protoperithecia production (middle panel), and lack of CAT fusion (lower panel) are shown for Δ*wsc-1;* Δ*ham-7*, and Δ*mak-1*.

The MAK-1 and MAK-2 MAP kinase signal transduction pathways have been shown to regulate hyphal anastomosis [15], [19], [20], [21]. To determine whether HAM-7 might function in one of the two pathways, we directly assayed the activity status of MAK-1 and MAK-2 in Δ*ham-7* (Figure 5A). Basal MAK-2 activity in Δ*ham-7* was reduced to 64%+/−17% of wild type (n = 5), while stress-induced levels were comparable to wild type, suggesting that MAK-2 function is not significantly affected in HAM-7. In contrast, the MAK-1 activity was clearly affected in Δ*ham-7*. Δ*ham-7* displayed reduced basal MAK-1 activity (15%+/−8% of wild type; n = 5), and stress-induction of MAK-1 was barely possible in Δ*ham-7*. Thus, HAM-7, like WSC-1, is required for activity of the *N. crassa* MAK-1 MAP kinase. We also tested if the function of the third MAP kinase pathway found in *N. crassa*, the OS-2 pathway, is altered in Δ*wsc-1* and Δ*ham-7*. We used antibodies directed against the phosphorylated phosphorylation site in OS-2 to assess the status of OS-2 in the deletion mutants and found that the Δ*ham-7* and Δ*wsc-1* mutants were not affected in activation of the OS-2 pathway (data not shown).

### A Δ*wsc-1;* Δ*ham-7* Double Mutant Fully Phenocopies Defects of MAK-1 Pathway Mutants

HAM-7 and WSC-1 are both required to activate MAK-1, but neither mutant fully phenocopies the Δ*mik-1*, Δ*mek-1* and Δ*mak-1* mutants that define the MAK-1 MAP kinase cascade [15], [16], [18]. These CWI pathway mutants share a common phenotype, which includes reduced vegetative growth, defective hyphal anastomosis (cell-to-cell fusion), a near total lack of macro- and microconidiation, and an inability to produce protoperithecia. Δ*wsc-1* phenocopies the reduced vegetative growth rate and conidiation defects seen in the Δ*mik-1*, Δ*mek-1*, and Δ*mak-1* mutants, but Δ*wsc-1* is able to undergo hyphal anastomosis and to form protoperithecia. Conversely, Δ*ham-7* phenocopies the Δ*mak-1* cell-to-cell fusion and protoperithecium defects, but clearly does not phenocopy the vegetative growth and conidiation phenotypes. Thus, the two sensors must function differently in regulating MAK-1 activation. We generated a Δ*wsc-1,* Δ*ham-7* double mutant to determine if the double mutant would fully mimic the phenotypic characteristic of MAK-1 pathway mutants. When we compared the double mutant with Δ*mak-1* the MAP kinase of the CWI pathway, we found that the two strains were morphologically very similar. Both strains produced conidiation-deficient, highly compact and rosetta-type colonies, were cell fusion defective and were unable to produce protoperithecia (Figure 5B, 5C). Moreover, MAK-1 activity levels were almost abolished in Δ*wsc-1;* Δ*ham-7* (Figure 5A). We conclude that WSC-1 and HAM-7 are the major sensors that function upstream of MAK-1, and that they regulate two distinct MAK-1 controlled cellular activities. WSC-1 regulates MAK-1 for the canonical CWI pathway, while HAM-7 activates MAK-1 during cell-to-cell fusion and female development.

## Discussion

The fungal cell wall is a dynamic structure that protects the cell from environmental stress. Moreover, the wall is critical for transmitting extracellular signals and in providing cell type-specific morphology. We have previously shown that mutations affecting the post-translation modification of cell wall proteins result in cell wall defects and morphological phenotypes in *N. crassa* [7], [8], [31], [42], [43]. One of the major findings of this work was that deletion mutants affecting the major “structural” cell wall proteins previously identified in a proteomic analysis of the *N. crassa* cell wall [8], [9] do not have defective cell walls based on our analysis. A large number of cell wall modifying enzymes that build the cross-linked glucan/chitin cell wall matrix were also included in our analysis, but none of the mutants were sensitive to the tested cell wall stress reagents nor were the mutants obviously defective in morphological characteristics. Maintaining the dynamics of the cell wall matrix is clearly important for the function of the fungal cell. Our results indicate a large level of “built-in redundancy” in cell wall biogenesis, and suggest that the loss of a single cross-linking enzyme can be compensated for by related enzymes with overlapping specificity.

We identified two cell wall proteins, HAM-7 and WSC-1 that are required for the formation of a normal cell wall and/or for other aspects of the *N. crassa* life cycle. Interestingly, WSC-1 and HAM-7 are both required for MAK-1 activity. However, Δ*ham-7* and Δ*wsc-1* show significant phenotypic differences that are characterized by a cell fusion/sexual developmental defect and a highly compact colony morphology and susceptibility to the glucan synthase inhibitor caspofungin, respectively.

The WSC family of proteins function as sensors for the cell wall integrity and stress response pathway in *S. cerevisiae* [13], [44] and in *Aspergillus* species [45], [46]. We suggest that *N. crassa* WSC-1 and its homolog WSC-2 function in a similar manner as sensors for the canonical CWI pathway. Δ*wsc-1* (and even more the Δ*wsc-1;* Δ*wsc-2* double mutant) closely resembles the vegetative growth characteristics of Δ*mik*-1, Δ*mek-1* and Δ*mak-1*, known mutants in the *N. crassa* cell wall integrity pathway [15], [16], suggesting that WSC-1 functions upstream of this pathway. This is especially evident in MAK-1 activity assays of the Δ*wsc-1;* Δ*wsc-2* double mutant, in which MAK-1 kinase activity is drastically reduced. Our mutant characterization demonstrates that WSC-1 is the major sensor that functions upstream of the CWI pathway in *N. crassa*, and that WSC-1 is especially important during vegetative growth and asexual development (i.e. the aerial hyphae formation and conidiation). The *N. crassa* genome harbors only two Wsc family genes, which is different from the situation in yeast, but similar to other filamentous ascomycetes [45], [46]. However, WSC-2 seems of minor importance for cell wall maintenance and the CWI pathway in *N. crassa*.

*S. cerevisiae* Wsc1p has been extensively characterized [12], [14]. *N. crassa* WSC-1 and WSC-2, like the yeast Wsc sensors, are single pass transmembrane proteins. Their extracellular domains have multiple serine, threonine, and arginine residues that could be sites for O- and N-glycosylation, similar to yeast Wsc1p where O-glycosylation results in a stiff rod-like structure. This region functions as nanospring and is thought to provide the protein with the ability to recognize cell wall stress [47]. The cytoplasmic tail of Wsc1p interacts with Rom2p, a GDP-GTP exchange factor that activates the GTPase Rho1p [44], which signals towards the MAPK cascade through activating Pkc1p [12], [14]. These elements are also present in the *N. crassa* genome [30] and function as part of the MAK-1 CWI pathway [15], [36], [37], [48]. The cytoplasmic tails of WSC-1 and WSC-2 show high levels of homology with the yeast proteins, while the extracellular domains are much less conserved, suggesting that the *N. crassa* sensors may receive distinct extracellular signals. Yeast Wsc1p has a cysteine-rich domain near the N-terminus, which has been implicated in clustering of the sensor [49]. The *A. fumigatus* WscA and WscB cell wall stress sensors also contain the cysteine-rich domain [45], while this domain is absent in *N. crassa* WSC-1.

The most prominent defects of Δ*ham-7* are its lack of cell fusion ability and the abolished formation of protoperithecia [22]. However, HAM-7 is also required for the morphology of the vegetative cell and for a normal branching pattern (Figure 2). Direct analysis of the levels of phosphorylated MAK-1 in Δ*ham-7* demonstrates that HAM-7 is required to activate MAK-1, but the mutant has no impact on MAK-2 activity (Fig. 3). This was an unexpected result, because vegetative cell communication and cell-to-cell fusion in *N. crassa* is thought to be primarily mediated through the NRC-1/MEK-2/MAK-2 MAP kinase module, which is homologous to the Ste11p/Ste7p/Fus3p mating pathway in budding yeast [15], [18], [19], [20], [50], [51]. Cell-to-cell fusion involves the oscillatory recruitment of the MAK-2 module and SO (a protein of unknown function) to the opposing tips of communicating pre-fusion cells. However, the MAK-1 pathway components MIK-1, MEK-1 and MAK-1 are also required for cell fusion [15], [17], and the relationship between the two MAP kinase pathways during hyphal fusion is currently unresolved. Fungal self-signaling is, so far, restricted to Pezizomycotina subphylum of ascomycetes [52], [53], which may explain why no *ham-7* homologs are detected in the genomes of unicellular ascomycetes and in basidiomycetes. Based on the fact that HAM-7 is an extracellular, GPI-anchored protein, it is likely that HAM-7 interacts with an unidentified transmembrane protein in order to activate a MAK-1 module required for cell-to-cell fusion. In summary, our data indicate that WSC-1 functions as the major sensor of a MAK-1 pathway similar to the canonical CWI pathway defined in *S. cerevisiae*, while HAM-7 plays a critical role in activating MAK-1 during cell-to-cell signaling and hyphal fusion.

## Supporting Information

Table S1 **List of the 65 cell wall proteins for which deletion mutants were available in the single gene deletion library.** The NCU numbers and names for the various proteins identified as cell wall proteins and potential cell wall proteins are based on the Broad Institute’s Neurospora genome website. The proteins identified via proteomics can be found in Maddi et al., 2009. Some additional proteins were then identified by GPI anchor predictors are found in De Groot et al., 2003 and in Eisenhaber et al. 2004. A few additional putative cell wall proteins were then identified by homology searches using protein sequences of known cell wall proteins from other fungi.
(DOC)

Table S2 **Primers for PCR amplification and cloning experiments.** The PCR primers used to clone wild-type genomic copies of the *wsc-1*, *ham-7*, and *acw-4* genes are shown. The underlined sequences are the restriction sites that were added to allow for the directional insertion of the PCR products into the pBM60 and pBM61 vectors.
(DOC)

Table S3 **Cell wall protein mutants with easily observed and/or stress-induced phenotypes.** Initial testing for stress sensitivities included growth at 37°C (heat stress), at 18°C (cold stress) in 10% NaCl (salt stress), in 0.05% mM H_2_O_2_ (peroxide stress), in 0.01% sodium dodecyl sulfate, in 2 M glycerol (osmotic stress), and in 10 µg/ml caspofungin acetate (glucan synthase inhibitor). Stress-induced growth defects as given as: N – normal growth (similar to wild-type cells), SL – slower growth, NG – no growth. Morphological characteristics observed for these mutants included the following: Protoperithecia defective (Proto-), reduced aerial hyphae formation (flat growth), hyphal tip lysis and conidial separation.
(DOC)
