# Supplementary material for: WSC-1 and HAM-7 Are MAK-1 MAP Kinase Pathway Sensors Required for Cell Wall Integrity and Hyphal Fusion in Neurospora crassa
Source: PLoS One. 2012 Aug 3;7(8):e42374. doi: 10.1371/journal.pone.0042374 (PMC3411791; doi:10.1371/journal.pone.0042374)
Supplement: Table S1 — List of the 65 cell wall proteins for which deletion mutants were available in the single gene deletion library. The NCU numbers and names for the various proteins identified as cell wall proteins and potential cell wall proteins are based on the Broad Institute’s Neurospora genome website. The proteins identified via proteomics can be found in Maddi et al., 2009. Some additional proteins were then identified by GPI anchor predictors are found in De Groot et al., 2003 and in Eisenhaber et al. 2004. A few additional putative cell wall proteins were then identified by homology searches using protein sequences of known cell wall proteins from other fungi. (DOC) [file pone.0042374.s001.doc]

**Table S1. List of the 65 cell wall proteins** for which deletion mutants were available in the single gene deletion library.

| **Protein Name/Description** | **Cell Wall defect** | **Gene Locus No.** | **Method used for Identification** |
| --- | --- | --- | --- |
| CHIT-1/Endochitinase (GPI-anchored) | Yes | NCU02184 | Proteomic analysis |
| NCW-1/Predicted protein | No | NCU05137 | Proteomic analysis |
| NCW-2/Predicted protein | No | NCU01752 | Proteomic analysis |
| NCW-3/Predicted protein | Yes | NCU07817 | Proteomic analysis |
| NCW-4/Predicted protein | No | NCU02948 | Proteomic analysis |
| NCW-5/Predicted protein | No | NCU00716 | Proteomic analysis |
| NCW-6/Predicted protein | No | NCU00586 | Proteomic analysis |
| ACW-1 (GPI-anchored) | No | NCU08936 | Proteomic analysis |
| ACW-2 (GPI-anchored) | No | NCU00957 | Proteomic analysis |
| ACW-3 (GPI-anchored) | No | NCU05667 | Proteomic analysis |
| ACW-5 (GPI-anchored) | No | NCU07776 | Proteomic analysis |
| ACW-6 (GPI-anchored) | No | NCU03530 | Proteomic analysis |
| ACW-8 (GPI-anchored) | Yes | NCU07277 | Proteomic analysis |
| ACW-9 (GPI-anchored) | No | NCU06185 | Proteomic analysis |
| ACW-11 (GPI-anchored) | No | NCU02041 | Proteomic analysis |
| GH16-7/Glycoside hydrolase (GPI-anchored) | No | NCU05974 | Proteomic analysis |
| β-glucosidase | No | NCU09326 | Proteomic analysis |
| GH17-3/Glucan- β-glucanase (GPI-anchored) | No | NCU09175 | Proteomic analysis |
| GH16-1/Mixed-linked glucanase/MLG (GPI-anchored) | No | NCU01353 | Proteomic analysis |
| GH72-5/GEL-1 (GPI-anchored) | Yes | NCU08909 | Proteomic analysis |
| GH55-3/GEL-2 (GPI-anchored) | No | NCU07253 | Proteomic analysis |
| GH72-2/GEL-5 (GPI-anchored) | No | NCU06781 | Proteomic analysis |
| CAT-3/Catalase | No | NCU00355 | Proteomic analysis |
| GH16-6/Chitin-glucan-cross linker | Yes | NCU05789 | Proteomic analysis |
| Predicted protein/Secreted protein | No | NCU00265 | Proteomic analysis |
| GH3-3/β-glucosidase 1 | No | NCU08755 | Proteomic analysis |
| ASD-1/ ascus development-1 | No | NCU05598 | Proteomic analysis |
| Predicted protein/Secreted protein | No | NCU04603 | Proteomic analysis |
| Predicted protein/Secreted | No | NCU00399 | Proteomic analysis |
| Predicted protein/Secreted | No | NCU08720 | Proteomic analysis |
| ACW-4 (GPI-anchored) | Yes | NCU09263 | Predicted GPI anchor |
| ACW-13 (GPI-anchored) | Yes | NCU01462 | Predicted GPI anchor |
| GH72-1/GEL-3 (GPI-anchored) | Yes | NCU01162 | Predicted GPI anchor |
| GH72-3/GEL-4 (GPI-anchored) | No | NCU06850 | Predicted GPI anchor |
| GH75-1/Chitosanase (GPI-anchored) | No | NCU02909 | Predicted GPI anchor |
| GH16-5/CGXL/Chitin-glucan-cross linker (GPI-anchored) | No | NCU05686 | Predicted GPI anchor |
| GH76-6/DCW1/5 (GPI-anchored) | No | NCU02216 | Predicted GPI anchor |
| GH76-8/DCW1/5 (GPI-anchored) | Yes | NCU00086 | Predicted GPI anchor |
| Alkaline protease (GPI-anchored) | No | NCU06055 | Predicted GPI anchor |
| HAM-7 (GPI-anchored) | Yes | NCU00881 | Predicted GPI anchor |
| Predicted protein (GPI-anchored) | No | NCU02884 | Predicted GPI anchor |
| Predicted protein (GPI-anchored) | No | NCU06109 | Predicted GPI anchor |
| Predicted protein (GPI-anchored) | No | NCU03873 | Predicted GPI anchor |
| Predicted protein (GPI-anchored) | No | NCU04496 | Predicted GPI anchor |
| Predicted protein (GPI-anchored) | No | NCU04542 | Predicted GPI anchor |
| Predicted protein (GPI-anchored) | No | NCU00473 | Predicted GPI anchor |
| Predicted protein (GPI-anchored) | No | NCU05229 | Predicted GPI anchor |
| Predicted protein (GPI-anchored) | No | NCU02170 | Predicted GPI anchor |
| Predicted protein (GPI-anchored) | Yes | NCU03222 | Predicted GPI anchor |
| Predicted protein (GPI-anchored) | No | NCU05395 | Predicted GPI anchor |
| Predicted protein (GPI-anchored) | No | NCU00322 | Predicted GPI anchor |
| Predicted protein (GPI-anchored) | No | NCU00175 | Predicted GPI anchor |
| GH16-13/Chitin-glucan-cross linker (GPI-anchored) | No | NCU00061 | Predicted GPI anchor |
| GH5-4/Exo-β-1-3-glucanase | No | NCU03914 | Homology search |
| SUN-4/Glucanase | No | NCU02668 | Homology search |
| GH81-1/DSE-4 | No | NCU07076 | Homology search |
| EGT-2/Endoglucanase | No | NCU01214 | Homology search |
| GH30-1/β-1,6-glucanase | No | NCU04395 | Homology search |
| Adhesin | No | NCU03773 | Homology search |
| PIR Mannoprotein | No | NCU04033 | Homology search |
| HKR/Osmosensor | No | NCU04373 | Homology search |
| WSC-1 | Yes | NCU06910 | Homology search |
| GH16-3/CGXL/Chitin-glucan-cross linker | No | NCU04431 | Homology search |
| Conserved hypothetical protein | No | NCU00711 | Homology search |
| Predicted secreted protein | No | NCU04675 | Homology search |

The NCU numbers and names for the various proteins identified as cell wall proteins and potential cell wall proteins are based on the Broad Institute’s Neurospora genome website. The proteins identified via proteomics can be found in Maddi et al., 2009. Some additional proteins were then identified by GPI anchor predictors are found in De Groot et al., 2003 and in Eisenhaber et al. 2004. A few additional putative cell wall proteins were then identified by homology searches using protein sequences of known cell wall proteins from other fungi.
