# Supplementary material for: WSC-1 and HAM-7 Are MAK-1 MAP Kinase Pathway Sensors Required for Cell Wall Integrity and Hyphal Fusion in Neurospora crassa
Source: PLoS One. 2012 Aug 3;7(8):e42374. doi: 10.1371/journal.pone.0042374 (PMC3411791; doi:10.1371/journal.pone.0042374)
Supplement: Table S2 — Primers for PCR amplification and cloning experiments. The PCR primers used to clone wild-type genomic copies of the wsc-1, ham-7, and acw-4 genes are shown. The underlined sequences are the restriction sites that were added to allow for the directional insertion of the PCR products into the pBM60 and pBM61 vectors. (DOC) [file pone.0042374.s002.doc]

Table S2: Primers for PCR amplification and cloning experiments.

| **Primer name** | **Primer sequence** |
| --- | --- |
| NCU06910 *wsc-1* forward (*NotI*) | TTAAGCGGCCGCTCCTTTCGTATCCGAACGCC |
| NCU06910 *wsc-1* reverse (*SpeI*) | ATTAACTAGTCTCTGAATGACGATGTCGACC |
| NCU00881 *ham-7* forward (*ApaI*) | AATTGGGCCCGCACATGGGTCATTTTGCCTGC |
| NCU00881 *ham-7* reverse (*EcoRI*) | AATTGAATTCCCAGATAAACGCCTAGTGGAC |
| NCU09263 *acw-4* forward (*BamHI*) | AATTGGATCCGCACAGCAGAGAGGC |
| NCU09263 *acw-4* reverse | CTGGCTGATGCAACCTTCTAC |

The PCR primers used to clone wild-type genomic copies of the *wsc-1*, *ham-7*, and *acw-4* genes are shown. The underlined sequences are the restriction sites that were added to allow for the directional insertion of the PCR products into the pBM60 and pBM61 vectors.
