# Supplementary material for: WSC-1 and HAM-7 Are MAK-1 MAP Kinase Pathway Sensors Required for Cell Wall Integrity and Hyphal Fusion in Neurospora crassa
Source: PLoS One. 2012 Aug 3;7(8):e42374. doi: 10.1371/journal.pone.0042374 (PMC3411791; doi:10.1371/journal.pone.0042374)
Supplement: Table S3 — Cell wall protein mutants with easily observed and/or stress-induced phenotypes. Initial testing for stress sensitivities included growth at 37°C (heat stress), at 18°C (cold stress) in 10% NaCl (salt stress), in 0.05% mM H2O2 (peroxide stress), in 0.01% sodium dodecyl sulfate, in 2 M glycerol (osmotic stress), and in 10 µg/ml caspofungin acetate (glucan synthase inhibitor). Stress-induced growth defects as given as: N – normal growth (similar to wild-type cells), SL – slower growth, NG – no growth. Morphological characteristics observed for these mutants included the following: Protoperithecia defective (Proto-), reduced aerial hyphae formation (flat growth), hyphal tip lysis and conidial separation. (DOC) [file pone.0042374.s003.doc]

**Table S3: Cell wall protein mutants with easily observed and/or stress-induced phenotypes.**

| **Protein/ Locus No.** | **37ºC** | **18ºC** | **NaCl**  **(10%)** | **H_2_O_2_**  **0.05mM** | **SDS**  **(0.01%)** | **Gly**  **(2M)** | **Caspo**  **(10μg/ml)** | **Morphology** | **Co-**  **segregation** |
| --- | --- | --- | --- | --- | --- | --- | --- | --- | --- |
| ACW-4  NCU09263 | SL | SL | NG | SL | NG | SL | NG | Flat growth,  Proto - | 24/24 |
| WSC-1  NCU06910 | N | N | N | N | N | N | NG | Slow, flat growth, aconidial | 24/24 |
| HAM-7  NCU00881 | SL | SL | N | SL | N | SL | N | Flat growth  Proto - | 24/24 |
| CHIT1  NCU02184 | SL | NG | NG | NG | NG | NG | NG | Slow growth,  Hyphal tip lysis | 19/94 |
| NCW-3  NCU07817 | SL | SL | NG | NG | NG | SL | NG | Flat growth,  Proto - | 18/24 |
| ACW-8  NCU07277 | N | N | SL | N | N | NG | N | Wild type | 9/24 |
| GEL-1  NCU08909 | N | N | NG | N | SL | SL | NG | Abnormal growth | 15/26 |
| GEL-3  NCU01162 | N | SL | NG | N | NG | SL | NG | Conidial separation | 14/23 |
| NCU03222 | N | N | NG | N | N | SL | NG | Wild type | 15/30 |
| GH16-6  NCU05789 | SL | NG | NG | SL | SL | N | SL | Flat growth,  Proto - | 13/24 |

Initial testing for stress sensitivities included growth at 37°C (heat stress), at 18°C (cold stress) in 10% NaCl (salt stress), in 0.05 mM H_2_O_2_, (peroxide stress), in 0.01% sodium dodecyl sulfate, in 2M glycerol (osmotic stress), and in 10 μg/ml Caspofungin acetate (glucan synthase inhibitor). Stress-induced growth defects are given as: N – normal growth (similar to wild-type cells), SL – slower growth, NG – no growth. Morphological characteristics observed for these mutants included the following: Protoperithecia defective (Proto -), reduced aerial hyphae formation (flat growth), hyphal tip lysis, and defective conidial separation:
